# The Dilemma of Postmodern Business Ethics: Employee Reification in a Perspective of Preserving Human Dignity

**DOI:** 10.3389/fpsyg.2022.813255

**Published:** 2022-02-15

**Authors:** Jolita Vveinhardt

**Affiliations:** Department of Management, Faculty of Economics and Management, Vytautas Magnus University, Kaunas, Lithuania

**Keywords:** employee reification, human dignity, moral relativism, catholic social teaching, business ethics

## Abstract

Management practices prevailing in business organizations receive considerable criticism for often treating the employee as one of many resources or an instrument to achieve the organization’s goals. As employee reification has so far been largely investigated in the scientific literature from the perspective of neo-Marxist approach, this article seeks to broaden the discussion by showing how social teaching of the Catholic Church can serve to solve the problem of reification. Although there is no doubt that universal norms of business ethics can serve as protection of the employee dignity from the individual’s reification tendencies, moral relativism operating in postmodern life tends to call into question any universal moral norms. Therefore, this article discusses how responses to challenges posed by moral relativism can be obtained by applying methodological approaches proposed by the neo-Marxist classics Lukács, Honneth, and Catholic Social Teaching. The similarities and differences of these approaches are identified, and attention is also drawn to the possibilities and limitations of their application in business ethics practice. It is also demonstrated how understanding of human dignity and the attitude to a virtue, offered by social teaching of the Catholic Church, broadens the discussion on addressing the dangers posed by the person’s reification in organizations.

## Introduction

In the last decade, the discourse on human resource management received considerable criticism for the prevailing attitude toward the employee (Islam, 2012; Rhodes and Harvey, 2012; Ombanda and K’Obonyo, 2019), as the individual’s instrumental treatment is encoded already in the very concept of the theory. For example, according to Drucker (1954), employees in principal differ from other enterprise resources in that they have “specific characteristics” and it is only them who can use themselves. That is, although the value of the employee is emphasized, the person is understood as a kind of thinking resource, personal motivation of whom determines how those “specific characteristics” will be used to achieve the goals of the organization. Therefore, the focus is on making the best use of such human resources by motivating, engaging them, encouraging communication, and this way seeking to maximize the effectiveness of the organization’s activities, which becomes a key function of the manager. This poses a risk of turning the employee into an instrument and an object of manipulation, bearing in mind the existing specific position of managerial power. Therefore, Rhodes and Harvey (2012) believe that human resource management cannot act as a moral guard of the organization’s activities merely because the ethics of labor relations are subordinated to the managerial prerogative. Alternatively, recognition of employee dignity and value in business ethics is proposed, which is often considered a significant means mitigating the negative consequences of the reifying treatment of an individual as a resource (Ashman and Winstanley, 2007; Islam, 2012; Arnaud and Wasieleski, 2014). Pirson (2019) in general proposes rethinking the objective function of the main management theory to create activities and wealth and recommends researchers to treat dignity as an inherent value and to focus on the protection of dignity and promotion of wellbeing or what is called the humanistic management theory.

Although it is proposed to adhere to ethics, often a complete answer as to why it should be followed is not provided. Should it be observed because it is beneficial for the organization to follow certain ethical norms or because it is “mandatory” and “moral” to do so for some circumstances? In one case, we have a certain utilitarian approach that brings us closer to the view that a person is an object or instrument which managers “must” treat appropriately for the common good of the organization because the employee has certain needs (e.g., a sense of dignity), the disregard of which will have an inappropriate effect resulting in losses. In the other case, the question arises as to what makes the behavior mandatory. Anscombe (1958) believes that utilitarianism does not protect from bad deeds and that talks about moral duty and what is “morally right or wrong” are meaningless when divine legislature is overstepped. In other words, there is a lack of a standard enabling to resolve the moral disagreements inherent in modern culture, because with the Age of Enlightenment, the context “in which moral judgments were understood as governed by impersonal standards justified by a shared conception of the human good” (MacIntyre, 2007, p. ix) was lost. Thus, speaking about business ethics, Walton (1993) emphasized the dilemma posed by postmodern philosophy: if there are no objective rules arising from human nature, then who determines them? In his opinion, there is no point of discussing business ethics until this question is answered. True, in this context, Gustafson (2000) motivated his critique of Walton by his wrongly represented postmodern philosophy as nihilistic and relativistic in itself. However, this does not make it clearer whether the understanding of corporate responsibility can be protected from subjective interpretation on the whole. On the other hand, it is observed that generally, the postmodern context of ethical life leaves no room for moral criticism, and this makes the society powerless against covert manipulations and oppression of powerful influential agents (Knappik, 2020). In solving this dilemma, one part of theorists turn their attention to religion-dictated principles of business ethics which would provide stability (Bay et al., 2010; Melé and Fontrodona, 2017; Bernacchio, 2019; etc.), while the other part develops the line of neo-Marxist approach that criticizes the attitude to the person as an instrument (Islam, 2012; Visser, 2019; etc.), emphasizing the person’s dignity. Although both perspectives pursue the same goals, the question remains to what extent the former is real in the secularizing society and to what degree the latter can be durable from the position of moral relativism.

This article is divided into four parts. The first and second parts present the attitudes of two neo-Marxist thinkers to the problem of reification of employees and interpersonal relationships, highlighting two quite different approaches. Due to the scope of the publication, the paper is limited to only two viewpoints, trying to show that although neo-Marxism is heterogeneous, theorists applying different approaches peculiarly complement each other. The third part of the article explains the basis of the attitude of Catholic Social Teaching to employee dignity and how this attitude can motivate religious leaders to adhere to ethical principles. The fourth part uses a quiz about moral relativism, enabling to check how business ethics grounded on different perspectives can remain durable in the postmodern reality. Finally, I discuss to what extent neo-Marxist and Catholic Social Teaching perspectives can be useful to business ethics.

## Reflection of Trade in Human Relationships in Lukács’ Critique

Criticism of the modern relation with employees and their “management” mostly focuses on the employee’s ethical (Greenwood, 2002) and exploitation aspects (Feenberg, 2011), and considerable attention to this problem is paid by the representatives of Frankfurt School of Philosophy, who develop the neo-Marxist doctrine. As early as in the third decade of the 20th century, Georg Lukács, a patriarch of this trend, identified reification (thing-making) of people and their interrelationships as an essential characteristic of the contemporary capitalist society. In what way are human relationships, and ultimately, people as well, turned into objects? Lukács pointed out that trade that takes place in the market was peculiarly repeated in human relationships. When most of good circulate as products, the initial natural relationships between producers and consumers are overshadowed. This gives rise to a new kind of society—the capitalist society. In that society, various relative qualities of objects and institutions are treated as things or as attributes of things (Karl Marx called them fetishes). According to him, prices determine production and move goods from one place to another, regardless of the value of their use. Corporations create reality, irrespective of the core activity through which they exist, while technical control is extended throughout the whole society, even to the individuals who use it (Lukács, 1971, p. 88–98). In Lukács’ view, the reality developed by corporations is repeated in human relationships and this way an unnatural, phantom reality contradictory to the natural order is created. This way, individuals and their relationships become reified, what Marx treated as alienation. He associated the retreat from the natural form of objectivity in social relationships, inherent in the medieval society, with the emergence of the capitalist society that perceived the social world as an effect of its actions (Feenberg, 2011). In this reality, rules, law, and like other institutes of social life lose their pre-industrial naturalness and are distorted, become mechanical, and adapt to the trade pattern (Hedrick, 2014; Varga, 2018). However, as Sitton (1998) emphasizes, neo-Marxist thinkers, such as Lukács and Habermas, believe that the boundaries of the market as a self-reproducing subsystem can only be drawn by a strict legal framework. In the words of Lukács himself, “the law maintains its close relationship with the ‘eternal values’” (Lukács, 1971, p. 109).

According to Tsogas (2018), p. 521, Lukács applies the principle of Kant’s philosophy, that is, “we can understand the things that we make, the ideas that we have, and the forms that we impose on reality.” In this case, only the proletariat can understand the reality of work, and when it understands that people are being turned into a commodity, it will rebel. Lukács associated awareness of the proletariat not only with resistance but also with a correct understanding of ethics. On the one hand, he linked the ethical consequences of actions with universal psychological facts, such as conscience and the sense of responsibility, stating that everyone bears personal responsibility and “from the ethical point of view, no one can escape responsibility with the excuse that he is only an individual, on whom the fate of the world does not depend” (Lukács, 2014, p. 8). That is, moral responsibility applies to everyone, regardless of the side of the ideological barricade on which the person stands. On the other hand, a morally fair action, according to him, “is related fundamentally to the correct perception of the given historicophilosophical situation, which in turn is only feasible through the efforts of every individual to make this self-consciousness conscious for himself.” Therefore, the growing awareness of employees becomes a decisive factor that turns into a precondition for changing the reified reality. However, in addition to awareness and responsibility, a significant place in Lukács’ ethics is occupied by common moral values. Nicolacopoulos and Vassilacopoulos (2020), p. 9 note that Lukács attributes to the ethical substantiality the power “to sustain and bind” the classless society of the future, in which love, understanding, and commonality become a moral foundation. In addition to that, such non-selfish human instincts as self-denial, sacrifice are transcendentalized and acquire absolute value (Kadarkay, 1994).

## Honneth’s Psychological Look at Human Dignity and Recognition

Axel Honneth, another neo-Marxist trend thinker, looked at the problem of reification through the psychological prism and human forgetfulness (Houston and Montgomery, 2017; Amaral and Hetti, 2020; Mookherjee, 2020) due to which human dignity suffers. As stated by Hedrick (2014), Honneth reconfigured the paradigm of reification, interpreting this as a basically interpersonal phenomenon that needed to be analyzed in the field of moral psychology. In this case, the theory of recognition comes to the fore, which, based on psychoanalytic insights, explains how a child emotionally recognizes the reality of another person significant to him, which is independent of the child’s own fantasies (Honneth, 1999). Honneth draws a parallel with the mechanism of recognition and engagement to others in the human developmental psychology. For example, if the child does not receive enough recognition, is unable to identify himself or herself with others emotionally, in the future, he or she will not be able to establish binding attachment relationships either with parents or with other persons and will not feel respect too. Thus, reification begins when people fail to empathize with other individuals (Ikechukwu and Jude, 2019). In other words, without empathy, the other person turns into a depersonalized object, and the relationships become socially pathological. Honneth derives the recognition itself from Hegel’s philosophy by extending it and distinguishing three kinds of harm or disrespect to a person: (1) doing physical harm, (2) social exclusion, and (3) downgrading of the social value of forms of self-realization (Honneth, 2001). Thus, this concerns recognition or non-recognition of physical and emotional needs. Because physical and emotional needs can, in a sense, be “confirmed” only by meeting or directly responding to them, recognition in this case acquires the nature of affective acceptance and encouragement. The relation of recognition itself is linked with the bodily existence of specific *Others* (people) who repay with a feeling of special respect (Honneth, 2001, p. 48).

He distinguishes a 3-fold model of recognition, consisting of love, legal order, and solidarity. All of it creates formal conditions for interaction, where people can be sure of their “dignity” and integrity. “Integrity” here only means that subjects can be calm, being aware that they are supported by the society. When they participate in social life in which they encounter all three models of recognition (in any form), they can positively relate themselves to the ways of self-confidence, self-respect, and self-esteem (Honneth, 2001, p. 50). On the one hand, according to Honneth, both the legal order and the community based on shared values are open to transformation processes directed to a higher degree of universality or equality. On the other hand, in addition to the absence of external pressure, there should be no internal psychological blockades, psychological inhibitions, and anxiety (Honneth, 2001, p. 51). Thus, it seems that Honneth does not tend to attach importance only to external social conditions but also takes into account the internal human barriers preventing realization.

How is human dignity reconcilable with the redistribution of goods, spoken of by classical Marxism, and with dignity and recognition? Honneth pointed out that a new social democratic idea emerged in the West in the ninth decade of the 20th century, the normative goal of which seemed to be not the elimination of inequality but the avoidance of degradation and disrespect; “equal distribution” or “equality of goods” no longer formed its central categories, but “dignity” and “respect” (Honneth, 2001, p. 43). According to him, “recognition” means mutual respect for the unique and equal status of all others; here, the behavior expected from discourse participants serves as a paradigm model (Honneth, 2001, p. 45). In this case, according to Honneth, a fair redistribution of goods is an integral part of recognition and respect, while disrespect is associated with the depreciation of the person’s contribution (Honneth, 1996, 1998, 2001). This aspect is important in business ethics, as recognition may lead the way for enterprises to adopt a caring stance for people and the surrounding environment and also helps to respond to the legitimate expectations of all groups in the society while conceiving themselves as an integral part of such society (Gold and Schleper, 2017). However, as Thompson (2014) observes, according to Honneth’s neo-idealist interpretation of socialization, the processes of self-development and ego formation take place outside these forces, but at the same time, initial child–parent relationships also become a channel for dominant value orientations and role expectations, which permeate culture. According to the author, Honneth’s model underestimates that recognition can also serve for the reproduction of forms of power, the heteronomous value systems.

## Employee Reification and Dignity in a Christian Perspective

Although the Christian perspective avoids the concept of reification while speaking about the depersonalized attitude to the employee, this coincides with a critique of treating the person as an object or “property” (Starck, 2004; Mosko, 2015). According to Bewes (2002), the term reification in Christianity may be metaphorically understood as Incarnation, but this concept differs from the Marxist tradition. In any case, the author calls the individual’s reification as a radical society’s secularization process that is both deistic and atheistic. In Christian anthropology, the treatment of the individual as a subject is derived from the person’s nature and has direct moral consequences. Because in the Christian tradition, God is treated as a person capable of making a respectful relation with another person who is created according to his image (Scott, 2006; Cochran, 2009; Spencer, 2018), this image exists in human interpersonal relationships and acts as an ethical source (Spencer, 2018). Similarly, Zagzebski (1998) links the motivation-based virtue theory with the divine person, who is the essential foundation of moral value. According to the author, “all moral concepts are derivative from the concept of a good motive,” which is a key component of virtue, initiating and directing action. Meanwhile, the belief that the person is created according to God’s image determines the fundamental approach that every human being is also endowed with the irreplaceable dignity that is the foundation of Catholic Social Teaching (Laczniak, 1999; Melé, 2011; Sison et al., 2016; Kovács, 2020). This approach essentially continues Thomas Aquinas’ idea that dignity means value that is determined by the inner essence of the “thing” itself, not by its usefulness (Brady, 2021). In other words, this sounds like an essential critique of business ethics insights in which recognition of the employee’s value stems from his/her value to the organization, since in the latter case, the person is treated as an instrument that needs good conditions to function well. In this case, a working environment that is favorable only to the employee or certain principles of “good” behavior do not satisfy the condition raised to the employer by Pope Leo XIII in his encyclical *Rerum Novarum*, which obliges “not to look upon their work people as their bondsmen, but to respect in every man his dignity as a person ennobled by Christian character” (Pope, 1891, p. 20). That is, without the approach to personal dignity, formed by Christian doctrine, teaching would remain quite a narrow set of approaches. In addition, between two flawed polar-opposite ideologies (Chicago Smith’s economic and socialism), Catholic Social Teaching offers an intermediate position in which “we find the virtue of economic justice where the individual’s natural but relative right to own property and his/her absolute dignity are guiding values” (Hühn, 2021, p. 13). At the same time, Hühn (2021) points to flexibility of teaching, which paves the way for discussions on business ethics issues.

According to Müller (2020), human dignity is understood not only as an anthropological principle meaning the intrinsic value of people but also as a practice-oriented foundation of morality, moral rights and obligations, and laws. It is not a man-made construct but a certain immutable law, which, according to Pastoral Constitution on the Church in the Modern World Gaudium et Spes of the Second Vatican Council, is discovered by people in the depths of their consciousness and which they must obey. However, this is also not a blind internal incentive or mere external pressure because the man’s dignity demands that he/she should act according to a knowing and free choice that is prompted and motivated from within (Gaudium et Spes, 1965). This presupposes personal responsibility because the perception of the person’s value gives rise to such relation with another person, corresponding to the human dignity, which can resemble only the relation with “the other self.” Therefore, “<…> disgraceful working conditions, where men are treated as mere tools for profit, rather than as free and responsible persons” (Gaudium et Spes, 1965) contradict human dignity, which is inseparable from both the rights to the common good created by the efforts of the whole community (Frémeaux and Michelson, 2017) and the person’s obligations. Sison et al. (2016) note that no one denies that work is also a right but insists that this should be perceived as an obligation. This is so because teaching on work-related rights appeared only in response to abuses in early industrialization; that is, later than the obligation itself. In the Judeo-Christian tradition, work as an obligation is inseparable from natural dignity (e.g., Gen 1:26, Gen 2:8, 15); thus, the employee cannot be treated merely as a “consumer” of the ethical relation with regard to his dignity; and he, in turn, becomes an equal partner with his dignified obligation. The employee and the manager meet as two persons performing dignified, albeit different, functions.

Another important factor is empathy, which integrates the theological concept of natural dignity in interpersonal relationships in practice. On the one hand, when the person expresses true empathy and concern for another person, he/she is not alienated in the relation with the true self (Tablan, 2016). On the other hand, the recognition of another person’s value and dignity substantiates the empathic relation with “the other self,” abandoning selfishness (Cremers, 2017), this way linking this attitude with interpersonal solidarity, which is one of the basic principles of Catholic Social Teaching (Beyer, 2014; Cremers, 2017; Mattison, 2018). Solidarity emphasizes the person’s social nature, linking the person’s right that his dignity is respected by others with the obligation to respect the dignity of others, which requires social responsibility from the individual (Cremers, 2017). According to Beyer (2014), although the concepts of solidarity can be found in other Christian traditions too, the Catholic social tradition has probably developed its conception to the fullest.

## Business Ethics in the Perspective of Moral Relativism

Postmodernism is closely related to the attitude to the relativity of ordinary moral principles. On the one hand, it is rooted in philosophical idealism (Bowden, 2019) and allows to assert the relativity of truth, at the same time providing a reason to write about organizations in general (Parker, 1995). On the other hand, as Eagleton (1996) argues, postmodernism is radical because it challenges the system that still needs absolute values while the result is, at best, an ingenious destruction of the dominant value system, at least at the theoretical level. In practice, this leads to the formation of a socially relativistic and narcissistic society, where morals and behaviors become subjective and individual, determining political and social fragmentation (Denton and Voth, 2017). In this case, it also makes sense to evaluate links existing between the person’s value approaches and psychological mechanisms. On the one hand, the emotional aspect in morality is emphasized by the doctrine of emotivism, which treats moral valuations as a kind of expression of feeling (MacIntyre, 2007). Because moral judgments are an expression of an attitude or feelings, according to MacIntyre, consensus on such decisions cannot be ensured by any rational methods. On the other hand, from the perspective of the Ethics Position Theory, the attitude to moral principles influences emotions, actions, while moral decisions are determined by the priorities given to relativism or idealism (Forsyth, 1980). According to Forsyth (1992), relativists believe that moral actions depend on the nature of the situation and the individuals involved, and, in valuing others, they weigh circumstances rather than consider ethical norms. Individuals prone to idealism, conversely, hold the view that it is necessary to act in such a way that actions correspond to moral principles or laws and do not have negative consequences for other people. The question therefore arises as to whether the ethical norm in business has sufficient power for persons who perceive ethics as a relative category in general?

Several studies conducted in recent years demonstrate that the tendency to rationalize ethical norms is related to the pursuit to ensure greater competitiveness and satisfaction of one’s own interests (Khan et al., 2019; Zaikauskaite et al., 2020). Although the neo-Marxist critique of capitalism accentuates the need for ethical standards to ensure that respect to employee dignity is observed in business practices, the problem of basic ethical principles becomes difficult to solve in the postmodern world, considering what is regarded as truth. Lukács admits that his ethics is “<…> tended in the direction of praxis,” which “led into economics, and the need for a theoretical grounding there finally brought me to the philosophy of Marxism” (Lukács, 1971, p. xi). Marx himself, according to Carver (2018), who reviewed his extensive creative legacy, despised socialists who paid much attention to ethics and rejected the moralizing discourse and universal principles. Therefore, in general, the foundation of the very concept of exploitation remains largely unclear. According to Ware (2019), those who considered him a moralist sometimes attributed to him universal principles but sometimes, relativistic principles that were associated with class or times.

It is debatable whether it is appropriate to strictly associate Marxists with moral relativism (for example, Ware (2019) argues that Marx did not have a formulated theory of morality but he followed moral positions), but Marxists’ belief that truth as well as fundamental ethical principles can be discovered by the mind itself cannot be denied (e.g., Lukács, 1971). Lukács associated this with the growth of the proletariat’s consciousness; therefore, the proletariat is perceived as a kind of competing power, the influence of which can fundamentally change the treatment of the man in the society. Lukács (1971) was convinced that the person’s reification could be overcome by way of the revolution initiated by the proletariat that has become conscious. Meanwhile, Honneth’s theory of recognition in itself demands the existence of universal moral principles, which he discovers in the essential human need to receive recognition in interpersonal relationships. Recognition requires an evaluation of the individual’s contribution to the society; however, he perceives the hierarchy of values, according to which this contribution is measured, as an outcome of a social agreement. This way the person’s recognition becomes dependent on the respect expressed by the society, which is acquired by means of symbolic force, seeking “to raise the value of the abilities associated with their way of life” (Honneth, 1996, p. 127). According to Smetona (2018), Honneth’s project consists of an attempt to completely abandon the Marxist nature of the reification concept as well as of the attempt to reconstruct the concept in purely normative terms as “forgetting” of intersubjective recognition, which he takes up.

Kim et al. (2009) state that Scripture-based Christian ethics provide business with moral standards enabling to judge what is right and wrong, although this does not mean that the moral limits of law and its various applications will not be discussed. However, according to the Thomistic tradition, these moral principles are related to “natural law,” which is discovered through its appeal to the “natural light of reason” (Dierksmeier and Celano, 2012). “Natural law” is perceived as a universal principle related to the human nature itself and is immutable, unlike the positive law, and “good is the first thing that falls under the apprehension of practical reason, which is directed to action: since every agent acts for an end under the aspect of good” (Aquinas, 1981, p. I–II 94).

The basic principle that the person discovers by his mind is that “good is to be done and pursued, and evil is to be avoided.” Therefore, “all other precepts of the natural law are based upon this: so that whatever the practical reason naturally apprehends as man’s good (or evil) belongs to the precepts of the natural law as something to be done or avoided” (Aquinas, 1981, p. I–II 94, 2). This attitude leaves no room for treating moral principles from the selfish personal perspective as it always directs to the fundamental principles developed in the doctrine of Catholic Social Teaching. Meanwhile, attempts to resolve ethical issues by renouncing God’s presence can lead to moral relativism when ethical standards are related to a particular culture, person, or historical time (Kim et al., 2009). The teaching of the Catholic Church distinguishes between subjective and objective treatment of work, which helps to avoid moral pluralism as regards the employee dignity. It is highlighted that work is, in an objective sense, the sum of activities, resources, instruments, and technologies used by men and women to produce things (Compendium of the Social Doctrine of the Church, 2004, p. 270). The subjective dimension is reflected in the fact that work is the activity of the man as a dynamic being who can perform various actions that are part of the work process and that correspond to his/her personal vocation. This aspect is stable because activities, technologies, and instruments (that is, the objective part of the work) are changing. This corresponds to human dignity because while working the man realizes his likeness to the Creator (Imago Dei). Any direct or indirect coercion, such as manipulation, violates human dignity. Therefore, the social teaching of the Catholic Church rejects that the man is only a “labour force,” another material means of production or an economic source. This means that the work not only stems from the person but also must be directed to him. In other words, work is for the man, but not the man is for work (Compendium of the Social Doctrine of the Church, 2004, p. 272). It is no coincidence that John Paul II in his encyclical announced on the occasion of the 90th anniversary of the encyclical of Pope Leo XIII “Rerum novarum” emphasized that the Church considered it her task always to call attention to the dignity and rights of those who work, and at the same time, not only to condemn situations in which that dignity and those rights are violated but also to try to influence processes in a positive direction (John, 1981).

## Discussion and Conclusion

Postmodern pluralism created conditions for legitimate existence of different approaches, which allows developing debates on the direction in which business ethics could evolve in order to better protect employee dignity from management practices violating it. Moral relativism spreading along with it limits the possibilities of applying universal ethical principles. Criticism of postmodern ethical life is related to the fact that no room is left for moral criticism in general, which makes the society powerless against covert manipulations and the oppression of powerful influential agents (Knappik, 2020). As noted by Poór et al. (2018), very relativistic individuals refuse universal moral principles, but non-relativistic persons adopt universal principles in making ethical decisions. Therefore, this article seeks to answer the question of how Catholic Social Teaching and neo-Marxist doctrines can do the quiz about moral relativism in business ethics.

On the one hand, there is considerable evidence that recognition of employees’ dignity can have a positive effect on the emotional state of employees themselves and on the relationship with the organization itself (Lucas et al., 2017; Thomas and Lucas, 2019; Noronha et al., 2020). Thus, as if there should be no questions regarding the value of recognizing employee dignity in management practices. On the other hand, however, the problem of the conception of human dignity itself emerges. For example, Lucas (2015) generally calls workplace dignity a phenomenon that is theoretically distinct from human dignity, while Kipper (2017) emphasizes the role of power imbalance, due to which dignity is not given to part of people. According to him, the violation of dignity arises due to mechanisms ensuring respect and attention for people who find themselves in an unfavorable situation. That is to say, the question arises as to whether it is possible to find objective criteria that would serve as a basis for drawing clear lines with regard to the attitude toward the employee as a person and what can ensure them. Attempts to provide the answer are made by quite heterogeneous neo-Marxist philosophical approaches. Although Lukács and Honneth approach the person’s reification from different positions, they not so much contrast with each other as complement one another. This shows that business ethics cannot be understood only as a totality of generally applicable rules because an important actor is the human nature and the psychological mechanisms dictated by it, which regulate interpersonal relationships. It can be envisaged that both neo-Marxist and Christian approach based on the philosophy of Thomas Aquinas share certain common ground, primarily in the belief in the existence of universal common human principles that can be discovered by the human mind. However, the Marxist tradition encounters a serious challenge that in Lukács’ case turns fair treatment of the employee into the game of power competition. Similarly, Honneth also makes the perspective too dependent on other persons’ attitudes and on the agreement of what will be considered human dignity. In both cases, the problem of moral relativism becomes difficult to overcome due to power gained by historical development processes and the struggle of different groups in the society. In this respect, the insight made by Kim et al. (2009) is important. The authors point out that in addition to moral relativism, modernism helped shape the meaning and purpose of one’s vocation or business, while the abandonment of the biblical approach to the creation means that humanity remained only a part of nature, guided by its interests and expedience. Therefore, another solution that allows to avoid different interpretations of the person’s dignity is proposed by Catholic Social Teaching.

Zigarelli (1993) notes that respect for the employee dignity, his or her family’s economic security, and the society’s wellbeing accentuated by Catholic Social Teaching clearly emerges as key guidelines for responsible human resource management, which could fundamentally change the nature of the enterprise’s ethics. In the opinion of other authors, such as Weaver and Agle (2002), internalized religious role expectations form the person’s religious identity and create the potential for religiously influenced ethical behavior. Such view is confirmed by some studies that find a link between religiosity and ethics violations in the regions with higher levels of religiosity of the population (Dyreng et al., 2012; McGuire et al., 2012). However, when enterprises operating in the environment of greater religiosity strive to act in a more responsible manner, the impact of the dynamics explained by the stakeholder theory also cannot be ruled out (Freeman, 2010). Involvement of stakeholder interests can strengthen enterprise’s moral commitments (e.g., Wes, 2009), thereby reducing the risks posed by moral relativism, but in this case, we cannot underestimate the problem posed by different teachings dictated by cultural diversity, which can be solved based on the internationally recognized human rights doctrine (Byrne, 2014). In this context, Tsogas (2018) is convinced that proper evaluation of Lukács’ reification theory can revise the approach to better international regulation of labor relations, which so far has not achieved positive results.

Of course, strong secularization trends taking place in the modern world are related to the fact that religiosity is increasingly becoming a “private sphere,” which leads to a certain separation of roles related to personal approaches and public behavior, that is, behavior in organizations. This may explain why some studies establish an ambiguous relationship between religiosity and business ethics (Parboteeah et al., 2008; Mazereeuw et al., 2014). This can be explained by the fact that the relationships between the religious role expectations and behavior are determined by the intensity of the religious identity and religious motivational orientation (Weaver and Agle, 2002). Nor can the criticism related to the religious persons’ attitude be underestimated. For example, Arli et al. (2020) argue that religiosity is related to ethnocentrism that can justify unethical behavior. However, this does not deny the strong motivating factor that promotes religious persons to consider ethical norms in practical activities more consistently (e.g., Zagzebski, 1998; Chowdhury, 2018; Spencer, 2018). In other words, community-recognized Christian moral norms can be an additional incentive for organizations to adhere to ethical norms, while personal religious beliefs influence the attitude toward another person in interpersonal relationships.

Although Marxist and Christian traditions use different terms, such as reification or instrumentalization (turning into an instrument to achieve goals), in principal they emphasize the individual’s depersonalization that violates human dignity and the person’s conversion into the object of manipulation. The common ground of neo-Marxist approach and the social teaching of the Catholic Church is treating employee reification or instrumentalization as dehumanization and the violation of human dignity. There is also general agreement on respect for the man, social guarantees corresponding to human dignity, and rights. However, views diverge on what is considered a source of human dignity. When dignity is inherently linked with a subjective psychological state, with what is agreed to be considered dignity, and when its protection is guaranteed by “fair” philosophical ethics and the distribution of goods based on it, significance is attached to ethical rules and laws of labor relations that should ensure the employee’s dignified position in organizations. However, ethical principles become a construct of the socio-philosophical thought and therefore succumb to a certain development of human thought. Thus, the rules, laws on ethics of labor relations may change. It is significant that from the Christian standpoint, in the changing conditions of history, contingent circumstances (such as technology and work tools), the value of the man cannot change. This is a sufficient basis for rejecting moral relativism and, thus, change in the ethics governing labor relations too, since human dignity derives from human nature with a divine spark, which does not change. Moreover, social teaching of the Catholic Church, according to Hühn (2021), is primarily based on the ethics of virtues, which requires doing the right things for the right reason in the right way and considering the situation. It is significant in this context that the ethical principles of social teaching of the Catholic Church become a virtue only by constantly repeating them in practice until they turn into the person’s internal approach in which the recognition of another person’s dignity comes to light, which does not allow to treat him/her as a tool (Guitián, 2015). Therefore, this mechanism can become an important basis in management practice while implementing leadership that selflessly focuses on employees (Shirin, 2015; Elche et al., 2020; Ruiz-Palomino et al., 2022). Because these virtues encompass all situations in which a person finds himself, life can only be such in which those virtues without which the man cannot attain his *telos* would fully unfold (MacIntyre, 2007). That is, adherence to ethical principles in the organization is driven by an internal approach that is supported by the *telos*-related pursuit of personal perfection as far as perfection is possible for the man. Marxism, meanwhile, proposes a collective approach, looking for an ethical solution through increasing the employees’ power. However, due to such involvement in the competition for power, the balance and flexibility offered by the social teaching of the Catholic Church are lost. Imposition of certain ethical norms or attitude to the person’s value from the position of power does not guarantee that such attitude will be followed consistently and honestly if moral approaches remain relative and dissociated from virtue.

## Author Contributions

The author confirms being the sole contributor of this work and has approved it for publication.

## Conflict of Interest

The author declares that the research was conducted in the absence of any commercial or financial relationships that could be construed as a potential conflict of interest.

## Publisher’s Note

All claims expressed in this article are solely those of the authors and do not necessarily represent those of their affiliated organizations, or those of the publisher, the editors and the reviewers. Any product that may be evaluated in this article, or claim that may be made by its manufacturer, is not guaranteed or endorsed by the publisher.
